# International Health Bureau

**Published:** 1920-02-21

**Authors:** 


### THE PUBLIC WELL-BEING.

### International Health Bureau.

### League of Nations' Decision.

An important step was taken on Friday by the Council of the League of Nations, sitting in St. James's Palace, and the decision reached followed the lines which The Hospital indicated last week—namely, the appointment of an expert body to advise on a permanent international health bureau. The proposals are clearly explained in the statement made by the Brazilian member of the Council; the points are appended.

The League of Nations, under articles of the League Covenant, is vested with very important functions relating to health matters throughout the world. One article states that the members of the League "will endeavour to take steps in matters of international concern for the prevention and control of disease" and another states that "the members of the League agree to encourage and promote the establishment and co-operation of duly authorised voluntary Red Cross organisations having as purposes the improvement of health, the prevention of disease, and the mitigation of suffering throughout the world."

If there is a field of action in which the League of Nations can bring immediate relief to nations and one which will affect individuals in their personal and family life, it is the field of social hygiene in the most liberal sense of the word. Health measures are essentially international measures, whether it be a question of adopting preventive or defensive means to combat contagious or epidemic diseases, or of popularising methods of cure and treatments. Without solidarity and an effective understanding between nations, any national organisation, however perfect in itself, will be incomplete. But neither the Council nor the Assembly of the League of Nations, nor the permanent Secretariat, possesses the requisite knowledge for the necessary technical consideration of scientific and social questions. What is necessary is a permanent organisation capable of co-ordinating, and drawing up the necessary statistics and keeping them constantly up to date. Without these no progress will be possible. This organisation should follow scientific research concerning public health, and should circulate its discoveries; it should co-ordinate and assist the action of organisations already existing, such as the Red Cross, the International Bureau of Public Health and other similar institutions; it should not only organise periodical and international conferences of scholars and health experts, but should call conventions similar to the Labour conferences. Finally, by a systematic propaganda, it should impress on public opinion the necessity of individual and collective rules and habits of health. Otherwise all conventions will be futile.

But to bring about the creation of this permanent organisation it would seem well to receive the proposals of a committee of competent authorities, instructed to submit proposals to this effect to the Council as soon as possible. The English Government, through its Health Office, has taken the initiative of calling together an informal commission with this in view, and this commission, with the addition of a small number of international health experts, and with an officer of the League as secretary, might be instructed by the Council to prepare the draft of a permanent international health organisation.

The following resolution was therefore submitted :—

"That in view of the duties imposed upon the League by Article 23 and Article 25, the Council invites the Health Commission, which has already met informally on the initiative of the British Government, to constitute a conference by adding to its members a small number of international health experts with an official of the League as secretary. The conference will prepare for submission to the Council proposals concerning the institution of a permanent body, to whom the Council can refer for advice and, if necessary, for action, all questions connected with the execution of the above-mentioned Articles."

The Council unanimously agreed.

